# Prospective Biomarkers from Plasma Metabolomics of Myalgic Encephalomyelitis/Chronic Fatigue Syndrome Implicate Redox Imbalance in Disease Symptomatology

**DOI:** 10.3390/metabo8040090

**Published:** 2018-12-06

**Authors:** Arnaud Germain, David Ruppert, Susan M. Levine, Maureen R. Hanson

**Affiliations:** 1 Department of Molecular Biology and Genetics, Cornell University, Ithaca, NY 14853, USA; ag297@cornell.edu (A.G.); cfssuelev@earthlink.net (S.M.L.); 2 Department of Statistical Science and School of Operations Research and Information Engineering, Cornell University, Ithaca, NY 14853, USA; dr24@cornell.edu

**Keywords:** ME/CFS, plasma, metabolomics, biomarkers, diagnostics

## Abstract

Myalgic Encephalomyelitis/Chronic Fatigue Syndrome (ME/CFS) is a disease of enigmatic origin with no established cure. Its constellation of symptoms has silently ruined the lives of millions of people around the world. A plethora of hypotheses have been vainly investigated over the past few decades, so that the biological basis of this debilitating condition remains a mystery. In this study, we investigate whether there is a disturbance in homeostasis of metabolic networks in the plasma of a female 32-patient cohort compared to 19 healthy female controls. Extensive analysis of the 832-metabolite dataset generated by Metabolon^®^, covering eight biological classes, generated important insight into metabolic disruptions that occur in ME/CFS. We report on 14 metabolites with differences in abundance, allowing us to develop a theory of broad redox imbalance in ME/CFS patients, which is consistent with findings of prior work in the ME/CFS field. Moreover, exploration of enrichment analysis using www.MetaboAnalyst.ca provides information concerning similarities between metabolite disruptions in ME/CFS and those that occur in other diseases, while its biomarker analysis unit yielded prospective plasma biomarkers for ME/CFS. This work contributes key elements to the development of ME/CFS diagnostics, a crucial step required for discovering a therapy for any disease of unknown origin.

## 1. Introduction

Myalgic Encephalomyelitis, also known as Chronic Fatigue Syndrome (ME/CFS) is a serious disease of unknown cause and poor prognosis. A number of studies have demonstrated abnormalities in the gut microbiome [1,2,3,4,5,6,7,8], the immune system [9,10,11,12], neuroimaging [13,14], exercise physiology [15], and blood metabolites [16,17,18,19,20,21,22], yet the underlying cause of the disease has not been identified [23,24]. Additionally, while data describing ME/CFS deficiencies continue to accumulate, there remains no explanation for the many symptoms of the disease.

We investigated the hypothesis that the homeostasis of metabolic networks in patients is disrupted when compared to healthy controls. Several studies, including from our lab, have already taken advantage of the fast-evolving field of mass spectrometry for metabolite identification and have explored metabolite composition of blood in several distinct populations [8,16,17,18,19,20,21]. A consensus is beginning to appear, with altered pathways being reported in multiple studies. Namely, several studies have established metabolism of lipids, oxidative stress and energy as relevant in ME/CFS. Unfortunately, no single or reasonably small set of metabolites have been determined to constitute basic metabolic signature of this disorder. Nevertheless, statistically significant differences in plasma or serum metabolites in multiple cohorts have been demonstrated using various metabolomics tools from a variety of expert teams. Differences between metabolite profiles of women and men with ME/CFS have also been observed [19].

In this report, we elaborate on 14 metabolites that we found to be different in abundance between our patient and healthy cohorts of females, and link this finding to a larger picture of metabolic pathway dysfunction. The highlighted pathways steer our understanding of the disease towards an effect on redox reactions, which has been a focal point in various studies [25,26,27,28,29,30]. We demonstrate that we can cross-validate our own studies with data from other investigators’ published reports. Our intent is to inspire deeper investigation into metabolomics in ME/CFS, with the goal of developing diagnostic tests.

## 2. Results

### 2.1. Cohort Statistics

The samples included in this study are both a subset of the population selected for our gut microbiome analysis and an expansion on the pilot cohort used in our first published metabolite analysis [21]. The final cohort is comprised of 19 controls and 32 patients, all female gender, with frequency-matched age and body mass index (BMI) between groups (Table 1). All patients selected meet the 1994 Fukuda definition [31] for ME/CFS, while the major criteria for the selection of controls was that they had no acute illnesses nor chronic fatiguing illnesses. This study was carried out in accordance with the recommendations of the Cornell University Institutional Review Board (CU IRB). The protocol (1012001855) was approved by the CU IRB. All subjects gave written informed consent in accordance with the Declaration of Helsinki.

The demographics statistics were similar between this current dataset, the Naviaux et al. dataset [19] and the one from Germain et al. [21] for both age and BMI. The population used for the Armstrong et al. [17] dataset was 15 years younger and no BMI information was provided.

### 2.2. Metabolite Data and Their Statistical Handling

In total, the Metabolon^®^ platform allowed identification and measurement of 832 metabolites across 51 subjects, for an aggregate of 42,432 data points. The classes of metabolites covered by this project include Amino Acids (177), Carbohydrates (26), Cofactors and Vitamins (28), Energy (10), Lipids (353), Nucleotides (29), Peptides (33), and Xenobiotics (176). These eight super-pathways, as defined by Metabolon^®^, can be further subdivided in 83 sub-pathways and this information can be found in our dataset provided as Supplemental File 1. While the names of most super-pathways are self-explanatory, it is relevant to note that the Xenobiotics category is comprised of metabolites belonging to the following sub-pathways: benzoate metabolism (11%), chemicals (14%), drugs (40%), food component/plant (24%), tobacco metabolism (2%) and xanthine metabolism (9%).

Metabolite values below the threshold of detection were dealt with by minimum value imputation for each biochemical within this project, as recommended by Metabolon^®^ and as common practice within the field of metabolomics. Missing values for drugs and tobacco metabolism, however, were not handled in such manner. It is logical that the lack of detection of these two metabolite classes reflects their actual absence, so that missing values were replaced by zero. Other metabolites occur in humans and therefore require an alternative method. There was a total of 5837 data points imputed, or 14% of the data. Drugs and tobacco metabolites represent 8% (3206) of the imputed data. While 513 metabolites did not require any data imputation, 156 metabolites had up to 20% of missing data, resulting in 543 imputations (1%). The remaining 90 metabolites had over 20% of missing data and 2088 (5%) imputations were performed.

Because 356 metabolites failed the Shapiro test of normality (*p* < 0.05) for at least one of the two groups (controls or patients) and a few severe outliers were present, it was concluded that the non-parametric Wilcoxon rank-sum test was more appropriate than a *t*-test, since the Wilcoxon test does not assume normally-distributed data and is not sensitive to outliers. To control the false discovery rate (FDR), Benjamini–Hochberg (BH) correction was applied to the *p*-values; the BH-adjusted *p*-values are often called *q*-values, terminology we have adopted. The BH adjustment was applied to the whole dataset as well as by Metabolon^®^ super-pathways; these are defined by Metabolon^®^ following their extensive review of literature but ultimately defined in house since there are obviously multiple pathways under which a compound may be categorized. Super-pathways result from the grouping of sub-pathways in accordance with their major feature, forming eight super-pathways as listed in the first section of results. Regarding sub-pathways, they result from the classification of metabolites in the major metabolic pathways to which they belong, as agreed by the metabolomics standards. This classification allows for broad biological interpretation of changes detected in metabolites belonging to the same super-pathway.

The non-parametric Wilcoxon rank-sum test was applied to our dataset and yielded 60 metabolites with significant differences at *p* < 0.05 (Table S1), or 7% of the metabolic networks assessed in this study. The geometric means of the patients/controls with a value lower than one is similar both for all metabolites and the 60-significant group at around 35%. Of note, 50% of the 60-significant group compounds belong to the lipid category and 15% to the amino acid category.

To adjust for multiple comparison testing, the Benjamini–Hochberg (BH) correction [32] was applied to the *p*-values to control the false discovery rate (FDR). When the BH correction was implemented, none of the *q*-values were below the threshold of *Q* < 0.15. However, when we applied the BH correction to each biological class of metabolites, following Metabolon^®^’s nomenclature, we obtained nine metabolites significantly different between controls and patients (Table 2).

### 2.3. The Metabolites Found to be Most Affected in ME/CFS Patients Belong to Four Classes

The nine metabolites discussed below belong to the following four super-pathways: “Cofactors and Vitamins”, “Energy”, “Nucleotides”, and “Peptides”. The Wilcoxon test returned *q* < 0.15 and *p* < 0.02 for those compounds (Table 2).

**Figure 1:**
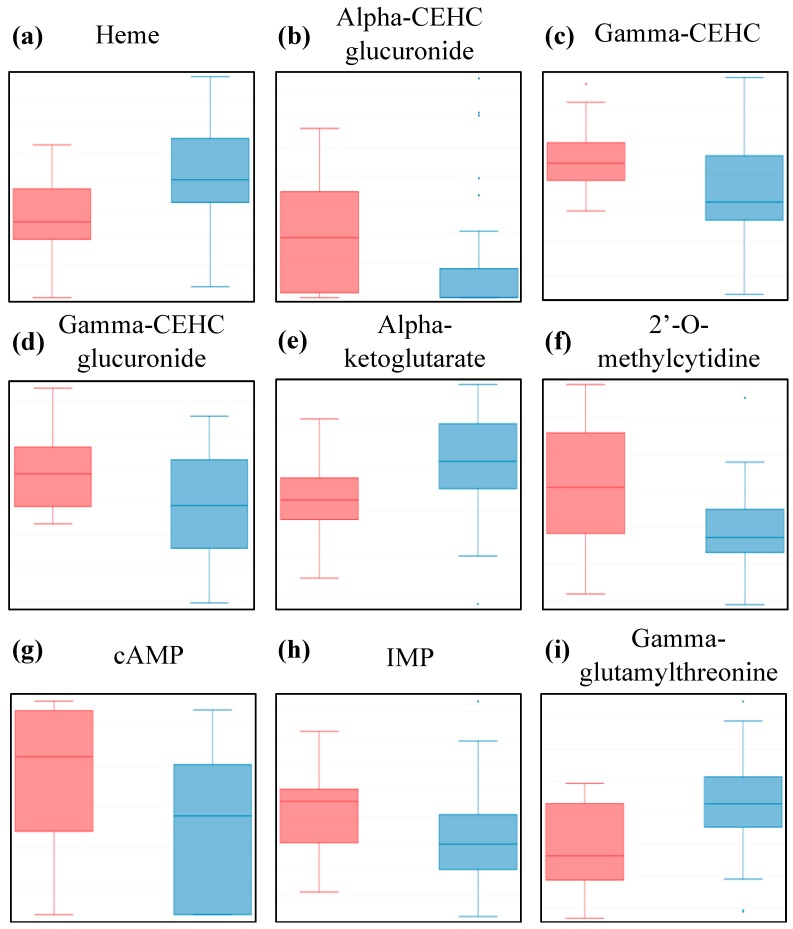
Box plot distribution of logged values for metabolites scored as being statistically different between controls (red) and patients (blue) at *p* < 0.05 and *q* < 0.15 by the Wilcoxon test. HMDB identity and test values can be found in Table 2. Y-axis scale is log10 transformed data.

Four of those metabolites are classified as “Cofactors and Vitamins”. Heme, the pigment that gives blood its red color, is one of the most statistically different metabolites, with higher abundance in patients compared to controls (Figure 1a and Table 2). Heme consists of an iron ion centered in a large organic ring and is mainly found in hemoglobin, but also in a few other important hemoproteins, with varying functions all related to redox chemistry. Free heme is highly cytotoxic and deleterious to tissues via its pro-oxidative and pro-inflammatory properties, as it is capable of catalyzing free radical formation [33,34,35].

The other three metabolites are part of the vitamin E pathway, and were significantly lower in patients compared to controls (Figure 1b—d, and Table 2). Vitamin E is a fat-soluble antioxidant that can hinder the propagation of reactive oxygen species through lipid membranes. Gamma-CEHC is the oxidized form of dietary gamma-tocopherol and is anti-inflammatory, while the other two are glucuronide conjugates of alpha-CEHC and gamma-CEHC, both significant circulating forms in the blood.

Another metabolite of interest is alpha-ketoglutarate, as it is one of the ten “Energy” metabolites for which we have data in this study. Our data indicates it is higher in patients compared to controls, (Figure 1e and Table 2). Alpha-ketoglutarate is an essential biological compound found in many biological pathways, linked to amino acid metabolism and part of the TCA cycle, which occurs in the mitochondria, where chemical energy is produced from the oxidation of pyruvate.

The following three metabolites belong to the “Nucleotides” class and all three have lower abundance in patients compared to controls (Figure 1f—h, and Table 2). Not much is known about 2’-O-methylcytidine, apart from the fact that it is part of the pyrimidine metabolism. On the other hand, adenosine 3’,5’-cyclic monophosphate (cAMP) and inosine 5’-monophosphate (IMP) are part of the purine metabolism and both associated with ATP/AMP, respectively, and are thus linked to energy metabolism. While the function of IMP in blood is still being discussed, cAMP is known to be a central intracellular regulator affecting hormonal pathways.

Finally, gamma-glutamyl-threonine, a dipeptide part of the “Peptides” class, was detected at significantly higher levels in the blood of patients compared to controls (Figure 1i and Table 2). Apart from the fact that this compound is an intermediate breakdown product of protein degradation, very little is known about its physiological effect in blood. It is, however, cited in a patent as a potential biomarker for liver toxicity determination [36].

### 2.4. The MetaboAnalyst Statistical Analysis Unit Provides Additional Valuable Insight into the Metabolon^®^ Dataset

The volcano plot tool combines fold changes and non-parametric testing for an alternative exploration of the data in order to bring in some biological significance to statistical analysis. A total of 7 metabolites stood out when using a fold change threshold of two and raw *p* < 0.05, while assuming an unequal group variance. Out of the 7, two overlap with the statistically significant metabolites described in the above section, namely heme and IMP.

All the other metabolites identified in the volcano plot had higher abundances in patients compared to controls (Figure S1). These included tauroursodeoxycholate (TUDCA), reported both as a cytoprotective agent and a chemical chaperone; 3-hydroxybutyrylcarnitine 1 and 3-hydroxybutyrate (BHBA), both involved in ketosis, a metabolic process associated with energy and glucose; piperine, an alkaloid found in herbs and spices; and histamine, a compound known to be involved in many aspects of the human body, including local immune responses, acting as a central neurotransmitter and a vasodilator to name a few.

### 2.5. The MetaboAnalyst Enrichment Analysis Unit Returns Compelling Disease-Associated Metabolite Sets in Plasma

The enrichment analysis tool allows similarity comparison between a query metabolite dataset, Metabolon^®^ in our case, and known disease-associated metabolite sets. This exploration was completed against a library of 344 human blood metabolite sets with a dataset limited to metabolites with a valid HMDB ID, 656 compounds out of the 832. While this tool identifies particular metabolites that vary similarly in other diseases to alterations detected in ME/CFS, it is not intended to be used for implying that all ME/CFS metabolite alterations are identical to those that occur in particular other diseases.

Ten metabolite sets had a *p* < 0.05 even though the *q*-values were not significant at 0.5 (Table 3). One of the intriguing patterns is that four out of the 10 diseases (40%) described, involve a dehydrogenase, oxidase or transferase deficiency. Those are all potentially linked to some redox state imbalance since it is highly unlikely that they are the result of gene mutations in the various affected pathways.

Because the statistical confidence from this module after correction is poor (*q* = 0.5), we performed the same analysis on published datasets, including our previous work, where such a tool had not been used. Such inquiries give us similar results, but with much stronger statistical power for some of them, displayed in Table 3. We include only the diseases in which at least half of the metabolite alterations were associated with the patient phenotype as the tool also returns results concerning alterations pertinent only to the differences in control metabolite levels. We have deliberately chosen not to discuss the particular metabolites used by this module, as it is not our intention to link ME/CFS to those diseases, but instead to observe potential trends in disease association. We discuss the potential links between ME/CFS symptoms and known physiological dysfunctions below.

Out of the ten metabolite sets from the Metabolon^®^ dataset, six were present in at least Armstrong et al. [17] or Germain et al. [21]. This shows strong commonality, a feature that we will further explore in result Section 2.7.

Our previous work [21] has abundant examples of dehydrogenase, carboxylase, hydrolase and transferase activity deficiencies, a few muscle-related conditions through variants of carnitine deficiencies, and some sugar related imbalances (Table 3 and data not shown). The Armstrong et al. dataset [17] also returns numerous dehydrogenase and carboxylase defects as disease-associated metabolite sets in blood (Table 3 and data not shown). Overall, dehydrogenase, transferase, and oxidase deficiencies are prevalent, and such a pattern leads us to suggest that a general imbalance impedes such type of reactions.

A notable condition found in the dataset analysis is anoxia (along with asphyxia) as this state is an extreme form of hypoxia, when the body, or a region of it, experiences extremely low oxygen. In a healthy person suffering from hypoxia, indicators include fatigue, confusion, headaches and numbness of extremities, which are all symptoms of ME/CFS patients. The oxygen status of tissue could possibly be affected by the disruption in heme abundance described in Figure 1a and Table 2. Reduced cerebral blood flow, which could result in inadequate brain oxygenation, has been hypothesized to be linked to cognitive impairment in ME/CFS patients [37,38].

Many diseases related to ketosis are also recurring in the analysis and are the result of an excessive buildup of ketones in the blood, a process mentioned above when discussing 3-hydroxybutyrate (BHBA) and 3-hydroxybutyrylcarnitine 1. For instance, diabetic ketoacidosis results from an inability to use carbohydrate for energy, causing metabolism of fats, thus implicating some disturbance in carbohydrate utilization in ME/CFS.

Finally, persistent hyperinsulinemic hypoglycemia of infancy (PHHI) was another disease found in two dataset results that can potentially be linked to brain activity [39]. Other diseases worthy of mentioning in regard to brain function were the correlations with metabolites found in schizophrenia, seizures and epilepsy, as well as several syndromes involving eye health. Notably, a common symptom in ME/CFS patients is extreme sensitivity to light.

### 2.6. The MetaboAnalyst Biomarker Analysis Unit Yields Prospective Biomarkers for ME/CFS Using Plasma

The machine learning feature used to illustrate the diagnostic ability is the receiver operating characteristic curve (ROC curve), which integrates true positive rates and false positive rates. The resulting graphical plot allows for the calculation of an area under the curve (AUC), where higher area values have a higher probability of accurate classification of a sample.

**Figure 2:**
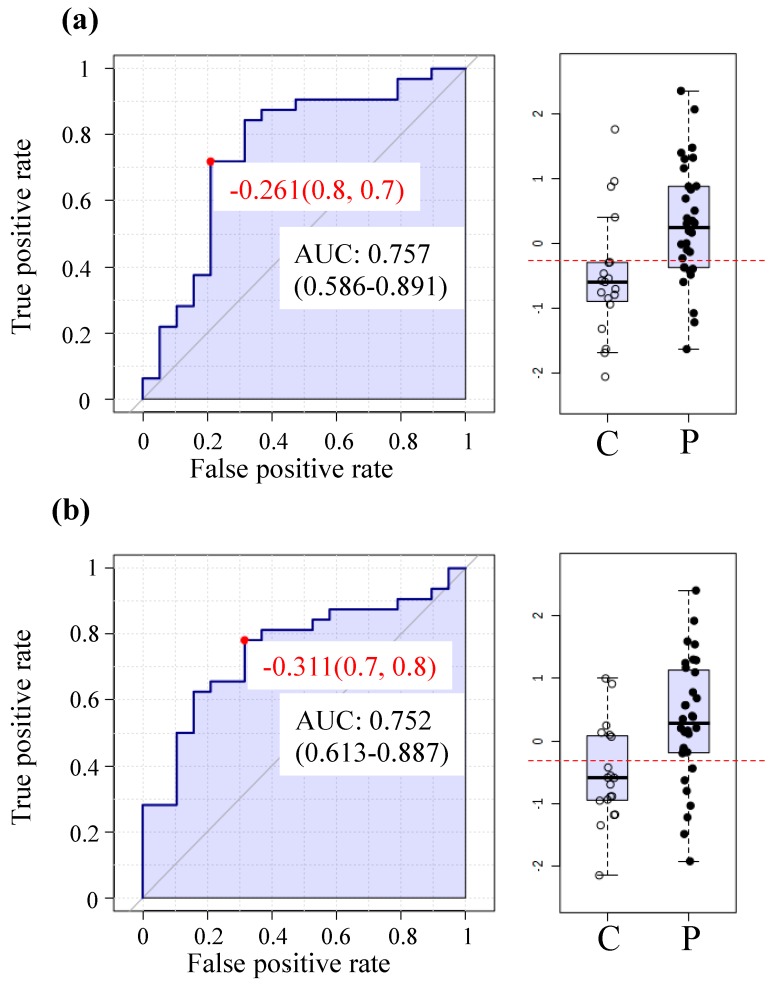
Receiver operating characteristic (ROC) curves and box plot distribution (C = controls; P = patients) for the top two highest prediction rates (**a**)—Pyroglutamine and (**b**)—Heme.

The highest prediction rates are achieved with pyroglutamine and heme with AUCs of 0.75 (Figure 2a,b respectively). 

Overall, 24 metabolites had AUC above 0.70 (Table S2) including heme, tauroursodeoxycholate, gamma-CEHC, gamma-CEHC glucuronide, IMP, alpha-ketoglutarate, while many of the other compounds are lipids.

### 2.7. Analysis Indicates that Close to 100% of the Cross-Identified Metabolites Exhibit Similar Statistical Behavior between Studies

The application of metabolomics to ME/CFS samples is still in an early stage, with only a few published studies. To review the reproducibility of results between research teams, with distinct populations and distinct metabolite identification technologies, we decided to perform pair comparisons. It should be noted that if the disease shows no effect in each of two studies, then our test using an interaction will be non-significant.

The first comparison was performed between our Metabolon^®^ dataset and Germain et al. [21], which used a smaller, but partially overlapping population on a different mass spectrometry platform. The comparison was implemented using 149 metabolites with the same HMDB identity. The null hypothesis that the mean difference (in log-abundance) between controls and patients is the same in both studies was tested. None of the metabolite behaviors were found to differ significantly between the two studies (Table 4).

We then proceeded to compare the complete Metabolon^®^ dataset to all of the data from Germain et al. [21]. In this case, 145 out of the 149 metabolites do not behave differently between either study, according to statistical analysis. The four exceptions (Table 4) are dihydrothymine, taurine, spermidine and acetylcarnosine, all of which were metabolites found to be significantly different between controls and patients in Germain et al. [21].

The next comparison was between the complete Metabolon^®^ dataset, and the female-gender dataset from Naviaux et al. [19]. Out of the 154 metabolites with common HMDB identities, only two, adenosine and flavin adenine dinucleotide (FAD), behave statistically differently between the two studies (Table 4). This difference could be due to the collection method of the plasma, which was done in EDTA for our samples while lithium-heparin tubes were used by Naviaux et al. [19].

Another comparison was made with a dataset of Armstrong et al. [17], and the complete Metabolon^®^ dataset. Even though only 26 metabolites had common HMDB IDs between both studies, 14 statistically behaved in the same manner (Table 4).

The final combinations included Germain et al. [21] and Armstrong et al. [17], Germain et al. [21] and Naviaux et al. [19], and Armstrong et al. [17] and Naviaux et al. [19], and the results were very similar, with 87%, 98% and 91% of metabolites, respectively, not behaving statistically differently between studies (Table 4).

Of note, is the recurring appearance of hypoxanthine as a metabolite found to be significantly different between studies, along with a few other metabolites, always involving comparisons with the Armstrong et al. dataset [17]. It is important to note that the Armstrong dataset was obtained by analysis of serum rather than plasma, which was used in all of the other studies. Even though both plasma and serum are sub-divisions of whole blood, significant differences in metabolite profiles have been shown to exist between the two [40].

### 2.8. Hierarchical Clustering Does not Yield any Definitive Subgroup Identification

One of the hypotheses concerning ME/CFS is that different fundamental disruptions occur in different sets of patients who happen to exhibit the same debilitating symptoms. The presence of patient subsets could explain why statistically significant differences between ME/CFS patients as a single group and a cohort of healthy individuals is sometimes not observed. The existence of subsets of patient types has also been suggested to explain why inconsistency between studies has been observed in some measures and why responses to treatment differ among patient populations [8,12,41,42,43,44].

A clustering analysis was performed on the Metabolon^®^ dataset in an attempt to identify potential differing metabolic signatures between two groups within the patient populations. No significant result was found when using either the full set of metabolites or the individual super-pathways (Table 5). The same conclusion was drawn when the analysis was implemented on the control group (data not shown).

Identical clustering analysis was performed on each of the other three datasets available, for which no super-pathway classification was available. Using patients only for Germain at al. [21], the *p*-values ranged from 0.940 to 0.954 and had a median of 0.946. For the Naviaux et al. dataset [19], the *p*-values ranged from 0.990 to 0.998 and had a median of 0.992. For Armstrong et al. [17], the *p*-values ranged from 0.008 to 0.032 and had a median of 0.020, making it the only dataset for which subgrouping was evident. To further investigate this finding, hierarchical clustering was applied to the Armstrong at al. dataset [17] using the R function hclust with the “complete” method. The dendrogram is displayed in Figure S2. We see that only two subjects (2 and 9) are somewhat separate from the rest; k-means clustering put these two patients in one cluster and the remaining patients in the second cluster, which would be only very weak evidence for subgrouping within the patient cohort. Using controls, no significant clustering was found in any of the three datasets (data not shown).

## 3. Discussion

The size of the population sampled in this study is comparable to published work such as Naviaux et al. [19] and Armstrong et al. [17], and is an expansion from the cohort we used in our previous effort [21], as displayed in Table 4. Moreover, the substantial number of metabolites quantified (832), is notably larger than the 420 compounds of Naviaux et al. [19], the 361 in Germain et al. [21] or the 29 in Armstrong et al. [17]. Undoubtedly, the statistical power of our analysis was weakened by the combination of a 51-subject population and an 832-metabolites array, explaining the limited number of metabolites we establish as significantly different in Table 2 and Figure 1, and only after super-pathway subgrouping. Nevertheless, our findings, through the extensive analysis of our dataset, shines light on an intriguing aspect of dysfunctional metabolism in ME/CFS patients, related to redox status.

Out of the four biological classes disturbed in our cohort, the “Nucleotides” and “Peptides” categories contain metabolites that potentially have broad repercussions on biological functions. For example, cAMP and IMP are compounds known to be involved in many aspects of human body function, such as purine metabolism, chemical energy storage in muscles, and intra-cellular signal transduction. It is therefore extremely difficult to pinpoint a singular pathway linked to ME/CFS status or symptoms based on such compounds or, on the contrary, using compounds of which there is little to no knowledge, such as 2’-O-methylcytidine or gamma-glutamylthreonine. The latter molecule, however, is mentioned as a potential compound of interest among many other biomarkers to determine liver toxicity of a given agent in a patent [36]. Results from our previous work [21] had focused attention on liver injury biomarkers.

Another metabolite of major interest is alpha-ketoglutarate because it is part of the “Energy” super-pathway and the TCA cycle sub-pathway. Indeed, the Krebs cycle is a pathway that consistently surfaces in ME/CFS metabolomics analysis across platforms and populations. Because fatigue is a major debilitating symptom of this disease, it has long been speculated that the energy metabolism of patients is dysfunctional. Several studies directly point to abnormal energy metabolism due to flawed TCA and urea cycles or directly upstream with putative impairment in pyruvate dehydrogenase [18,20]. A pilot study using a patented nutraceutical treatment hypothesized to boost the activity of this enzyme, and consequently the Krebs cycle, describes substantial improvements to the health and condition of treated patients [45]. Nevertheless, alpha-ketoglutarate is involved in numerous metabolic pathways such as carnitine metabolism, lysine metabolism and branched-chain amino acids, to name a few, so that a focus on a single pathway as the foundation of the disabling symptoms of ME/CFS is presently unjustified.

The “Cofactors and Vitamins” category encompasses metabolites with disparate properties, as exemplified by heme and gamma-CEHC. Higher levels of heme, part of the “Hemoglobin and Porphyrin metabolism”, and lower levels of gamma-CEHC, part of the “Tocopherol metabolism”, were measured in ME/CFS patients compared to controls in our cohort (Figure 1). Heme is a vital component of many metalloproteins, the most well-known being hemoglobin, and is synthesized in the liver and the bone marrow. As the Metabolon^®^ sample preparation is methanol-based, protein precipitation is expected even though protein-bound heme could still be released depending on the level of heme coordination. Because we used plasma, which is a cell-free matrix, it is anticipated that there is a greater contribution from “free heme” to the measurement of heme abundance unless substantial hemolysis occurred. High concentration of free heme in plasma is a biomarker for sickle cell disease severity, in which increased levels of inflammatory biomarkers such as lactate dehydrogenase, bilirubin, high reticulocytes count, and lipids are detected [35]. Three forms of bilirubin as well as biliverdin were assessed in our samples and were also present in higher abundance in patients vs. controls, demonstrating a general disturbance in the heme degradation pathway, with the last step occurring in the liver. All five compounds have strong deleterious effects that are linked to free radical generation and their degradation is claimed to be part of a cytoprotective feedback in response to oxidative stress [46]. Antagonistically, gamma-CEHC, along with gamma-CEHC glucuronide and alpha-CEHC glucuronide, are metabolites of the vitamin E pathway, which has anti-inflammatory features, such as acting as a lipophilic antioxidant [47]. Our previous work also suggested a disruption in vitamin E metabolism as a result of detection of 13’-carboxy-alpha-tocopherol [21], which is unfortunately not present in this dataset.

Many ME/CFS patients self-report specialized diets as well as supplements as management tools to mitigate their symptoms. Such behavior is widespread within the patient communities of many diseases, including ME/CFS, fibromyalgia and cancer to only name a few [48,49,50]. Excluding prescription pharmaceuticals, such nutrition approaches can either be defined by dietary restrictions or associated with specific supplements thought to exhibit beneficial effects against inflammation, cardiovascular problems or aging. The adoption of restrictive diets to ameliorate a potential wheat sensitivity in some patients [51] or ketogenic diets and fasting, have been reported to be helpful by other patients [48]. Many patients also consider some supplements beneficial, including NADH, coenzyme Q10 or polyphenols, although systematic review of study outcomes does not lead to clear recommendations to the patient community [52,53]. However, a commonality between all the work on nutrition in ME/CFS cited above can be found in redox metabolism. Clearly, our current work as well as other reports suggests that nutritional alterations might be of assistance to patients, though further research is necessary before any recommendations can be made.

An ambitious aim in applying untargeted metabolomics to ME/CFS samples is to probe for a pattern that can further our limited understanding of this disease. The enrichment analysis unit of MetaboAnalyst revealed a potential imbalance in the redox state of patients, as their metabolic profiles matched several conditions unrelated to each other, but which all involved redox enzymatic reactions (Table 3). Our hypothesis is that a disturbance in the redox status influences the status of chemical reaction donors and acceptors as well as their coenzymes such as NAD+/NADH, FAD+/FADH for dehydrogenases. Oxidases would obviously also be affected as catalyzers of redox reactions.

Many transferase catalytic activities could be influenced by the redox state of their environment and many illnesses are caused by transferase deficiencies. For instance, succinyl CoA: 3-ketoacid CoA transferase deficiency leads to a buildup of ketones and diabetic ketoacidosis [54], as reported in Figure S1b and S1c and Table 3 respectively. Carnitine palmitoyl transferase deficiency II (CPT II) is another example in which fatty acid metabolism is disrupted by the lack of transport of long chain fatty acids into the mitochondria, where they are used as a fuel source [55].

Anoxia is one of the disease-associated metabolite sets shown in Table 3, a condition that also appeared using the Armstrong et al. datasets [17] (data not shown), and along with asphyxia in the Germain et al. dataset [21] (data not shown). The ME/CFS metabolite profile also has similarities to those of infants who develop hypoxic-ischemic encephalopathy (HIE) due to oxygen deprivation. Anoxia and asphyxia are both linked to the lack of oxygen, which can obviously have severe repercussions on muscle and body activity. Inability to deliver oxygen to muscles adequately is evident in studies of response to exercise in ME/CFS patients [15,56,57]. Furthermore, prefrontal cortex oxygenation of the brain is reduced in exercising ME/CFS patients [58]. Many individuals with ME/CFS exhibit reduced blood volume, thus affecting oxygenation of many tissues [59,60]. Disturbances in circulation and provision of oxygen to tissues could underlie many symptoms of ME/CFS. Hypoxia results in generation of reactive oxygen species by mitochondria, resulting in activation of protective systems [61].

An association of oxidative stress and ME/CFS has been reported in a number of prior ME/CFS studies [24,25,26,27,28,29]. Of note, when measuring known oxidative stress markers, Richards et al. [25] found that methaemoglobin was one of the principal components that differentiated their ME/CFS patients and control cohorts. This hemoglobin carries the oxidized form of the iron ion, namely, the ferric state instead of the ferrous state necessary for the hemoglobin to bind oxygen. Even though methaemoglobin measurements are not part of our dataset, it is intriguing to relate its oxidation state to a disturbed redox environment while the effect of the inability to bind oxygen could translate into anoxia and asphyxia.

ME/CFS biomarkers, as a mean of unambiguous diagnosis and monitoring of efficacy of therapies, are one of the urgently needed developments in this field. Figure 2 as well as Table S2 were generated from univariate ROC analysis with this goal in mind. This method used metabolite to accomplish the highest prediction rates with over 0.75 AUC. Future work in which a larger and independent cohort is analyzed and compared to other fatiguing illnesses will likely increase the prediction confidence and will reveal whether plasma metabolomics may serve as a reliable tool for objective identification and monitoring of ME/CFS patients.

## 4. Materials and Methods 

### 4.1. Sampling and Metabolomics Platform

Blood samples were collected in NYC by ME/CFS expert physician Dr. Susan Levine in EDTA tubes and shipped overnight by FedEx in a Styrofoam box to Cornell University-Ithaca Biotechnology Building, where plasma was separated from cells by centrifugation at 500 g for 30 min, before being stored at −80 °C until further analysis.

Plasma samples were shipped frozen on dry ice to Metabolon^®^, where Global Metabolomics was performed using four ultra-high-performance liquid chromatography/tandem accurate mass spectrometry (UHPLC/MS/MS). This automated service allows accurate relative quantification of hundreds of metabolites spanning wide categories of compounds. Extensive details can be found in Supplementary Material and Methods.

### 4.2. Data Analysis through MetaboAnalyst

Our dataset was processed through MetaboAnalyst 4.0 [62], a comprehensive tool for metabolomics analysis and interpretation (www.metaboanalyst.ca). For each module used, data was uploaded either as a complete dataset with the chemical name or as a restricted list of HMDB IDs when required. As recommended for untargeted metabolomics datasets, data filtering was applied in the following order: interquantile range (IQR), relative standard deviation (RSD = SD/mean) and non-parametric relative standard deviation (MAD/median. Abundance data was uploaded as a csv file in unpaired rows, and normalization was achieved by log transformation and auto scaling (mean-centered and divided by the standard deviation of each variable).

### 4.3. Data Analysis in-House

The metabolite abundances were log-transformed using natural logarithms, but the test results would be identical if base-10 logarithms had been used.

Comparisons were made for each pair of studies. For each pair, an analysis of variance was performed on each metabolite with log-abundance as the response and Disease (patient or control) and Study (Study 1 or Study 2) as factors. The *p*-value for the interaction between patients and controls can be used to test the null hypothesis that the difference in log-abundances between patients and controls is the same in the two studies.

K-means clustering analysis was executed on all datasets available to us. For our dataset, k-means clustering was applied separately to each of the 10 super-pathways using the R function kmeans (with centers = 2 and nstart = 10). This means that two cluster were to be found and the algorithm was started 10 times to better locate well-separated clusters. Only patients were used and metabolite abundances were log-transformed. The ratio of the between-clusters sum of squares to the total sum of squares was used as a test statistic (see below for calculation of *p*-values) and called the “SSratio”. For each super-pathway, 250 normally-distributed datasets were generated, each of size equal to the number of patients. The simulated datasets had the same mean and covariance as the patients’ log-abundances for that pathway. Since their mean and covariance matrix were the same for all simulated observations, the simulated datasets had no subgroups. The *p*-value for a super-pathway was the number of SSratios from the simulated datasets that exceeded the SSratio of the actual data. Because k-means clustering and the simulation of datasets are random algorithms, the *p*-value was calculated five times for each super-pathway in the Metabolon^®^ data. For the other datasets, no super-pathway separation was available. To reduce the number of variables, the only metabolites used were those where the ratio of the mean for patients to the mean for controls was either above 1.1 or below 0.9. The selected metabolites were then log-transformed and a *p*-value was calculated as with the Metabolon^®^ dataset. *p*-values were computed 7 times, each with 500 simulated datasets.

### 4.4. Data Availability

The dataset generated in this study is available for download as Supplementary File 1.

## 5. Conclusions

In this study, we report on untargeted metabolomics profile of a relatively small cohort. Despite statistical challenges, we provide yet more evidence for a redox imbalance in ME/CFS patients. The rationale behind our theory is bolstered by parallels with our previous study and available datasets from other teams. The patient cohorts that have been used in our studies and others differ in geographical location, diet, treatment regimes, yet there are remarkable similarities among the findings. Thus, metabolomics may be revealing a fundamental feature of the disruption that occurs in victims of ME/CFS.

## Figures and Tables

**Table 1:** Population statistics. The Bell scale is reported for patients who answered the survey, with 71% indicating substantial impairment in function (10–40).

		Controls	Patients
**Gender**	Female	19	32
**Age**	Mean +/− SD	49.2 +/− 10.5	48.5 +/− 13.7
	Median (range)	50 (20–66)	50 (19–71)
**BMI**	Mean +/− SD	26.8 +/− 5.9	25.5 +/− 5.7
	Median (range)	26.1 (17–41)	24.35 (16–41)
**Bell’s Disability Scale**	10–20	ND	7
	30–40		13
	50–60		5
	>60		3
	N/A		4
**Onset of Illness**	Gradual	ND	17
	Sudden		15
**Duration of Illness**	<3 years	ND	6
	>3 years		26
**Intestinal Discomfort**	Yes	ND	23
	No		9

N/A stands for Not Answered. ND stands for Not Determined.

**Table 2:** List of metabolites found to be significantly different between controls and patients according to the Wilcoxon rank-sum test.

Super-Pathway	Metabolites	HMDB ID	Wilcoxon Rank-Sum Test	
			*p*-Value	*q*-Value
**Cofactors and Vitamins**	Heme	HMDB03178	0.002	0.06
	Gamma-CEHC	HMDB01931	0.005	0.08
	Alpha-CEHC glucuronide	HMDB62445	0.018	0.13
	Gamma-CEHC glucuronide	N/A	0.019	0.13
**Energy**	Alpha-ketoglutarate	HMDB00208	0.003	0.03
**Nucleotide**	Inosine 5’-monophosphate (IMP)	HMDB00175	0.003	0.11
	2’-O-methylcytidine	N/A	0.009	0.13
	Adenosine 3’-5’-cyclic monophosphate (cAMP)	HMDB00058	0.012	0.13
**Peptide**	Gamma-glutamylthreonine	HMDB29159	0.003	0.11

All metabolites with a *q* < 0.15 are included. N/A stands for Not Assigned. HMDB stands for Human Metabolome Database.

**Table 3:** List of disease-associated metabolite sets in blood for the following datasets: Metabolon^®^ (M^®^), Armstrong et al. [17] and Germain et al. [21].

Metabolite Set	Dataset	Total	Hits	*p*-Value	*q*-Value
**Long-Chain-3-Hydroxyacyl-CoA Dehydrogenase Deficiency (LCHAD)**	M^®^	10	2	0.01	0.5
	[17]	10	2	0.0005	0.004
	[21]	-	-	-	-
**Anoxia**	M^®^	8	4	0.02	0.5
	[17]	-	-	-	-
	[21]	8	6	0.23	0.33
**Diabetic Ketoacidosis**	M^®^	2	1	0.02	0.5
	[17]	-	-	-	-
	[21]	-	-	-	-
**Obesity**	M^®^	2	1	0.02	0.5
	[17]	-	-	-	-
	[21]	-	-	-	-
**Persistent Hyperinsulinemic Hypoglycemia of Infancy (PHHI)**	M^®^	3	1	0.02	0.5
	[17]	3	1	0.001	0.002
	[21]	-	-	-	-
**Succinyl CoA:3-ketoacid CoA Transferase Deficiency**	M^®^	3	1	0.02	0.5
	[17]	-	-	-	-
	[21]	-	-	-	-
**Heart Failure**	M^®^	10	3	0.02	0.5
	[17]	-	-	-	-
	[21]	-	-	-	-
**Pyridoxamine 5’-Phosphate Oxidase Deficiency**	M^®^	3	2	0.04	0.6
	[17]	-	-	-	-
	[21]	3	2	0.55	0.65
**Carnitine Transporter Defect. Primary Systemic Carnitine Deficiency**	M^®^	4	1	0.05	0.6
	[17]	4	1	0.001	0.002
	[21]	-	-	-	-
**Carnitine Palmitoyl Transferase Deficiency II (CPT II)**	M^®^	8	4	0.05	0.6
	[17]	8	1	0.001	0.002
	[21]	8	3	0.03	0.75

The metabolite sets selected have a *p* < 0.05 for the analysis of the Metabolon^®^ dataset. The “total” column reflects the number of known metabolites affected by a specific disease while the “hits” column reflects the number of metabolites similarly affected in the patient cohort.

**Table 4:** Study pair comparisons with cohort size.

Study 1	Study 2	*q*-Value < 0.15
**Metabolon^®^**	**Germain et al. [21]**	None
Identical population (13 controls & 9 patients)		
**Metabolon^®^** 19 controls & 32 patients	**Germain et al. [21]** 18 controls & 18 patients	5,6-dihydrothymine, taurine, spermidine, N-acetylcarnosine
**Metabolon^®^** 19 controls & 32 patients	**Naviaux et al. [19]** 21 controls & 23 patients	adenosine, flavin adenine dinucleotide (FAD)
**Metabolon^®^** 19 controls & 32 patients	**Armstrong et al. [17]** 25 controls & 34 patients	glucose, glutamate, hypoxanthine, phenylalanine, alanine, proline, threonine, asparagine, lysine, serine, lactate, creatinine
**Germain et al. [21]** 18 controls & 18 patients	**Armstrong et al. [17]** 25 controls & 34 patients	glucose, glutamate, hypoxanthine
**Germain et al. [21]** 18 controls & 18 patients	**Naviaux et al. [19]** 21 controls & 23 patients	gluconate, S-1-pyrroline-5-carboxylate
**Armstrong et al. [17]** 25 controls & 34 patients	**Naviaux et al. [19]** 21 controls & 23 patients	hypoxanthine, lactate

HMDB IDs listed correspond to metabolites that a significant difference between the two studies in the ratio of abundance in patients to abundance in controls at *q* < 0.15.

**Table 5:** Clustering Analysis *p*-values.

Name	Patients		
	Median	Minimum	Maximum
**All Metabolites**	0.980	0.980	0.992
**Amino Acids**	0.992	0.988	1.000
**Carbohydrates**	0.976	0.968	0.988
**Cofactors and Vitamins**	0.500	0.432	0.552
**Energy**	0.216	0.208	0.264
**Lipids**	0.936	0.900	0.952
**Nucleotides**	0.624	0.596	0.632
**Peptides**	0.764	0.744	0.808
**Xenobiotics**	0.728	0.708	0.736

The analysis was done on the complete dataset (All metabolites), and each super-pathway for the patient cohort.

## Supplementary Materials

The following are available online at http://www.mdpi.com/2218-1989/8/4/90/s1, Figure S1: Box plot distribution of logged values for metabolites scored as being statistically different between controls (red) and patients (blue) according to the volcano plot tool from MetaboAnalyst. (**a)**—HMDB00874; (**b)**—HMDB13127; (**c)**—HMDB00357; (**d)**—HMDB29377; (**e)**—HMDB00870. Y-axis scale is log10 transformed data, Figure S2: Dendrograms resulting from the hierarchical clustering of the Armstrong et al. dataset [17]. The samples displayed are all patients and the numbering is consistent with the original dataset, Table S1: List of metabolites found to be significantly different between controls and patients according to the Wilcoxon test. *p* < 0.05 are shown in bold. N/A stands for Not Assigned. * reflects metabolites for which no reference spectrum was needed to identify the compound. [1] and [2] refers to isomers of the same metabolite. The geomean ratio column reports the ratio of the geometric means of the patients/controls, Table S2: List of metabolite ratios with AUC above 0.70 resulting from ROC curves predictions, with *t*-tests values and log2 of fold-change (FC).

## References

[1] Borody T., Nowak A., Finlayson S. (2012). The GI microbiome and its role in chronic fatigue syndrome: A summary of bacteriotherapy. J. Nutr. Envron. Med..

[2] Fremont M., Coomans D., Massart S., De Meirleir K. (2013). High-throughput 16S rRNA gene sequencing reveals alterations of intestinal microbiota in myalgic encephalomyelitis/chronic fatigue syndrome patients. Anaerobe.

[3] Shukla S.K., Cook D., Meyer J., Vernon S.D., Le T., Clevidence D., Robertson C.E., Schrodi S.J., Yale S., Frank D.N. (2015). Changes in gut and plasma microbiome following exercise challenge in myalgic encephalomyelitis/chronic fatigue syndrome (ME/CFS). PLoS ONE.

[4] Giloteaux L., Goodrich J.K., Walters W.A., Levine S.M., Ley R.E., Hanson M.R. (2016). Reduced diversity and altered composition of the gut microbiome in individuals with myalgic encephalomyelitis/chronic fatigue syndrome. Microbiome.

[5] Giloteaux L., Hanson M.R., Keller B.A. (2016). A pair of identical twins discordant for myalgic encephalomyelitis/chronic fatigue syndrome differ in physiological parameters and gut microbiome composition. Am. J. Case Rep..

[6] Armstrong C.W., McGregor N.R., Lewis D.P., Butt H.L., Gooley P.R. (2017). The association of fecal microbiota and fecal, blood serum and urine metabolites in myalgic encephalomyelitis/chronic fatigue syndrome. Metabolomics.

[7] Hanson M.R., Giloteaux L. The gut microbiome in myalgic encephalomyelitis. http://www.biochemist.org/bio/03902/0010/039020010.pdf.

[8] Nagy-Szakal D., Williams B.L., Mishra N., Che X., Lee B., Bateman L., Klimas N.G., Komaroff A.L., Levine S., Montoya J.G. (2017). Fecal metagenomic profiles in subgroups of patients with myalgic encephalomyelitis/chronic fatigue syndrome. Microbiome.

[9] Brenu E.W., van Driel M.L., Staines D.R., Ashton K.J., Ramos S.B., Keane J., Klimas N.G., Marshall-Gradisnik S.M. (2011). Immunological abnormalities as potential biomarkers in chronic fatigue syndrome/myalgic encephalomyelitis. J. Transl. Med..

[10] Morris G., Berk M., Galecki P., Maes M. (2014). The emerging role of autoimmunity in myalgic encephalomyelitis/chronic fatigue syndrome (ME/CFS). Mol. Neurobiol..

[11] Nijs J., Nees A., Paul L., De Kooning M., Ickmans K., Meeus M., Van Oosterwijck J. (2014). Altered immune response to exercise in patients with chronic fatigue syndrome/myalgic encephalomyelitis: A systematic literature review. Exerc. Immunol. Rev..

[12] Hornig M., Montoya J.G., Klimas N.G., Levine S., Felsenstein D., Bateman L., Peterson D.L., Gottschalk C.G., Schultz A.F., Che X. (2015). Distinct plasma immune signatures in ME/CFS are present early in the course of illness. Sci. Adv..

[13] Boissoneault J., Letzen J., Lai S., O’Shea A., Craggs J., Robinson M.E., Staud R. (2016). Abnormal resting state functional connectivity in patients with chronic fatigue syndrome: An arterial spin-labeling fMRI study. Magn. Reson. Imaging.

[14] Gay C.W., Robinson M.E., Lai S., O’Shea A., Craggs J.G., Price D.D., Staud R. (2016). Abnormal resting-state functional connectivity in patients with chronic fatigue syndrome: Results of seed and data-driven analyses. Brain Connect.

[15] Keller B.A., Pryor J.L., Giloteaux L. (2014). Inability of myalgic encephalomyelitis/chronic fatigue syndrome patients to reproduce VO(2)peak indicates functional impairment. J. Trans. Med..

[16] Armstrong C.W., McGregor N.R., Sheedy J.R., Buttfield I., Butt H.L., Gooley P.R. (2012). NMR metabolic profiling of serum identifies amino acid disturbances in chronic fatigue syndrome. Clin. Chim. Acta.

[17] Armstrong C.W., McGregor N.R., Lewis D.P., Butt H.L., Gooley P.R. (2015). Metabolic profiling reveals anomalous energy metabolism and oxidative stress pathways in chronic fatigue syndrome patients. Metabolomics.

[18] Fluge O., Mella O., Bruland O., Risa K., Dyrstad S.E., Alme K., Rekeland I.G., Sapkota D., Rosland G.V., Fossa A. (2016). Metabolic profiling indicates impaired pyruvate dehydrogenase function in myalgic encephalopathy/chronic fatigue syndrome. JCI Insight.

[19] Naviaux R.K., Naviaux J.C., Li K.F., Bright A.T., Alaynick W.A., Wang L., Baxter A., Nathan N., Anderson W., Gordon E. (2016). Metabolic features of chronic fatigue syndrome. Proc. Nat. Acad. Sci. USA.

[20] Yamano E., Sugimoto M., Hirayama A., Kume S., Yamato M., Jin G.H., Tajima S., Goda N., Iwai K., Fukuda S. (2016). Index markers of chronic fatigue syndrome with dysfunction of TCA and urea cycles. Sci. Rep..

[21] Germain A., Ruppert D., Levine S.M., Hanson M.R. (2017). Metabolic profiling of a myalgic encephalomyelitis/chronic fatigue syndrome discovery cohort reveals disturbances in fatty acid and lipid metabolism. Mol. Biosyst..

[22] Nagy-Szakal D., Barupal D.K., Lee B., Che X., Williams B.L., Kahn E.J.R., Ukaigwe J.E., Bateman L., Klimas N.G., Komaroff A.L. (2018). Insights into myalgic encephalomyelitis/chronic fatigue syndrome phenotypes through comprehensive metabolomics. Sci. Rep..

[23] Navaneetharaja N., Griffiths V., Wileman T., Carding S.R. (2016). A role for the intestinal microbiota and virome in myalgic encephalomyelitis/chronic fatigue syndrome (ME/CFS)?. J. Clin. Med..

[24] Gerwyn M., Maes M. (2017). Mechanisms explaining muscle fatigue and muscle pain in patients with myalgic encephalomyelitis/chronic fatigue syndrome (ME/CFS): A review of recent findings. Curr. Rheumatol. Rep..

[25] Richards R.S., Roberts T.K., McGregor N., Dunstan R.H., Butt H.L. (2000). Blood parameters indicative of oxidative stress are associated with symptom expression in chronic fatigue syndrome. Redox. Rep..

[26] Maes M., Kubera M., Uytterhoeven M., Vrydags N., Bosmans E. (2011). Increased plasma peroxides as a marker of oxidative stress in myalgic encephalomyelitis/chronic fatigue syndrome (ME/CFS). Med. Sci. Monitor..

[27] Arnett S.V., Clark I.A. (2012). Inflammatory fatigue and sickness behaviour—lessons for the diagnosis and management of chronic fatigue syndrome. J. Affect Disord..

[28] Jammes Y., Steinberg J.G., Delliaux S. (2012). Chronic fatigue syndrome: acute infection and history of physical activity affect resting levels and response to exercise of plasma oxidant/antioxidant status and heat shock proteins. J. Intern. Med..

[29] Morris G., Maes M. (2014). Oxidative and nitrosative stress and immune-inflammatory pathways in patients with myalgic encephalomyelitis (ME)/chronic fatigue syndrome (CFS). Curr. Neuropharmacol..

[30] Fenouillet E., Vigouroux A., Steinberg J.G., Chagvardieff A., Retornaz F., Guieu R., Jammes Y. (2016). Association of biomarkers with health-related quality of life and history of stressors in myalgic encephalomyelitis/chronic fatigue syndrome patients. J. Transl. Med..

[31] Fukuda K., Straus S.E., Hickie I., Sharpe M.C., Dobbins J.G., Komaroff A. (1994). The chronic fatigue syndrome: A comprehensive approach to its definition and study. International Chronic Fatigue Syndrome Study Group. Ann. Intern. Med..

[32] Benjamini Y., Hochberg Y. (1995). Controlling the false discovery rate—a practical and powerful approach to multiple testing. J. Roy. Stat. Soc. B. Met..

[33] Schaer D.J., Buehler P.W., Alayash A.I., Belcher J.D., Vercellotti G.M. (2013). Hemolysis and free hemoglobin revisited: exploring hemoglobin and hemin scavengers as a novel class of therapeutic proteins. Blood.

[34] Chiabrando D., Vinchi F., Fiorito V., Mercurio S., Tolosano E. (2014). Heme in pathophysiology: A matter of scavenging, metabolism and trafficking across cell membranes. Front. Pharmacol..

[35] Carvalho M.O.S., Rocha L.C., Reis J.H.O., Santos T.D., do Nascimento V.M.L., Carvalho M.B., Luz N.F., Borges V.D., Goncalves M.S. (2015). Heme concentration as a biomarker of sickle cell disease severity: Its role in steady-state and in crisis patients. Blood.

[36] Milburn M., Guo L., Wulff J.E., Lawton K.A. (2014). Determining liver toxicity of an agent using metabolite biomarkers. U.S. Patent.

[37] Biswal B., Kunwar P., Natelson B.H. (2011). Cerebral blood flow is reduced in chronic fatigue syndrome as assessed by arterial spin labeling. J. Neurol. Sci..

[38] Staud R., Boissoneault J., Craggs J., Lai S., Robinson M.E. (2018). Task related cerebral blood flow changes of patients with chronic fatigue syndrome: an arterial spin labeling study. Fatigue.

[39] Ninkovic D., Sarnavka V., Basnec A., Cuk M., Ramadza D.P., Fumic K., Kusec V., Santer R., Baric I. (2016). Hyperinsulinism-hyperammonemia syndrome: A de novo mutation of the GLUD1 gene in twins and a review of the literature. J. Pediatr. Endocr. Met..

[40] Liu L.S., Aa J.Y., Wang G.J., Yan B., Zhang Y., Wang X.W., Zhao C.Y., Cao B., Shi J.A., Li M.J. (2010). Differences in metabolite profile between blood plasma and serum. Anal. Biochem..

[41] Maes M., Leunis J.C., Geffard M., Berk M. (2014). Evidence for the existence of myalgic encephalomyelitis/chronic fatigue syndrome (ME/CFS) with and without abdominal discomfort (irritable bowel) syndrome. Neuroendocrinol. Lett..

[42] Guenther S., Loebel M., Mooslechner A.A., Knops M., Hanitsch L.G., Grabowski P., Wittke K., Meisel C., Unterwalder N., Volk H.D. (2015). Frequent IgG subclass and mannose binding lectin deficiency in patients with chronic fatigue syndrome. Hum. Immunol..

[43] Hornig M., Gottschalk C.G., Eddy M.L., Che X., Ukaigwe J.E., Peterson D.L., Lipkin W.I. (2017). Immune network analysis of cerebrospinal fluid in myalgic encephalomyelitis/chronic fatigue syndrome with atypical and classical presentations. Transl. Psychiat..

[44] Scheibenbogen C., Loebel M., Freitag H., Krueger A., Bauer S., Antelmann M., Doehner W., Scherbakov N., Heidecke H., Reinke P. (2018). Immunoadsorption to remove beta 2 adrenergic receptor antibodies in chronic fatigue syndrome CFS/ME. PLoS ONE.

[45] Comhaire F. (2017). A novel nutriceutical treatment of myalgic encephalitis/chronic fatigue syndrome (ME/CFS): “What it is and what it is not”. Intern. Med..

[46] Chen W.Y., Maghzal G.J., Ayer A., Suarna C., Dunn L.L., Stocker R. (2018). Absence of the biliverdin reductase-a gene is associated with increased endogenous oxidative stress. Free Radical. Bio. Med..

[47] Wu X.L., Beecher G.R., Holden J.M., Haytowitz D.B., Gebhardt S.E., Prior R.L. (2004). Lipophilic and hydrophilic antioxidant capacities of common foods in the United States. J. Agr. Food Chem..

[48] Craig C. (2015). Mitoprotective dietary approaches for myalgic encephalomyelitis/chronic fatigue syndrome: Caloric restriction, fasting, and ketogenic diets. Med. Hypotheses..

[49] Lopez-Rodriguez M.M., Molina J.G., Medina I.M.F., Sola C.F., Muelle A.R. (2017). Patterns of food avoidance and eating behavior in women with fibromyalgia. Endocrinol. Diab. Nutr..

[50] McCubrey J.A., Lertpiriyapong K., Steelman L.S., Abrams S.L., Yang L.V., Murata R.M., Rosalen P.L., Scalisi A., Neri L.M., Cocco L. (2017). Effects of resveratrol, curcumin, berberine and other nutraceuticals on aging, cancer development, cancer stem cells and microRNAs. Aging.

[51] Uhde M., Indart A.C., Yu X.B., Jang S.S., De Giorgio R., Green P.H.R., Volta U., Vernon S.D., Alaedini A. (2018). Markers of non-coeliac wheat sensitivity in patients with myalgic encephalomyelitis/chronic fatigue syndrome. Gut.

[52] Campagnolo N., Johnston S., Collatz A., Staines D., Marshall-Gradisnik S. (2017). Dietary and nutrition interventions for the therapeutic treatment of chronic fatigue syndrome/myalgic encephalomyelitis: A systematic review. J. Hum. Nutr. Diet..

[53] Jones K., Probst Y. (2017). Role of dietary modification in alleviating chronic fatigue syndrome symptoms: A systematic review. Nutrition.

[54] Fukao T., Sass J.O., Kursula P., Thimm E., Wendel U., Ficicioglu C., Monastiri K., Guffon N., Baric I., Zabot M.T. (2011). Clinical and molecular characterization of five patients with succinyl-CoA:3-ketoacid CoA transferase (SCOT) deficiency. Bba-Mol. Basis Dis..

[55] Sigauke E., Rakheja D., Kitson K., Bennett M.J. (2003). Carnitine palmitoyltransferase II deficiency: A clinical, biochemical, and molecular review. Lab. Invest..

[56] Vermeulen R.C.W., Kurk R.M., Visser F.C., Sluiter W., Scholte H.R. (2010). Patients with chronic fatigue syndrome performed worse than controls in a controlled repeated exercise study despite a normal oxidative phosphorylation capacity. J. Transl. Med..

[57] Snell C.R., Stevens S.R., Davenport T.E., Van Ness J.M. (2013). Discriminative validity of metabolic and workload measurements for identifying people with chronic fatigue syndrome. Phys. Ther..

[58] Neary J.P., Roberts A.D.W., Leavins N., Harrison M.F., Croll J.C., Sexsmith J.R. (2008). Prefrontal cortex oxygenation during incremental exercise in chronic fatigue syndrome. Clin. Physiol. Funct. I.

[59] Streeten D.H.P., Thomas D., Bell D.S. (2000). The roles of orthostatic hypotension, orthostatic tachycardia, and subnormal erythrocyte volume in the pathogenesis of the chronic fatigue syndrome. Am. J. Med. Sci..

[60] Streeten D.H.P., Bell D.S. (2011). Circulating blood volume in chronic fatigue syndrome. Fatigue.

[61] Smith K.A., Waypa G.B., Schumacker P.T. (2017). Redox signaling during hypoxia in mammalian cells. Redox. Biol..

[62] Chong J., Soufan O., Li C., Caraus I., Li S., Bourque G., Wishart D.S., Xia J. (2018). MetaboAnalyst 4.0: Towards more transparent and integrative metabolomics analysis. Nucleic Acids Res..

